# Sources of Fungal Symbionts in the Microbiome of a Mobile Insect Host, *Spodoptera frugiperda*

**DOI:** 10.1007/s00248-022-02140-3

**Published:** 2022-12-08

**Authors:** Monica Watson, Georgiana May, Kathryn E. Bushley

**Affiliations:** 1 Graduate Program in Ecology, Evolution & Behavior, University of Minnesota, St. Paul, MN USA; 2 Department of Ecology, Evolution & Behavior, University of Minnesota, St. Paul, MN USA; 3 Emerging Pests and Pathogens Unit, USDA-ARS, Ithaca, NY USA

**Keywords:** Microbiome, Symbiont, Fungi, Insect, *Spodoptera frugiperda*, *Sorghum*, Vertical and horizontal transmission

## Abstract

The sources of fungal symbionts of insects are not well understood, yet the acquisition and assembly of fungal communities in mobile insect hosts have important implications for the ecology of migratory insects and their plant hosts. To determine potential sources of fungi associated with the fall armyworm (*Spodoptera frugiperda*), we characterized the fungal communities associated with four different ecological compartments (insects, infested leaves, uninfested leaves, and soil) and estimated the contributions of each of these potential sources to the insect’s fungal microbiome. Results show that insect fungal community composition was distinct from and more varied than the composition of fungal communities in the environment of those insects (plants and soil). Among the sources evaluated, on average we found a surprisingly large apparent contribution from other congeneric *S. frugiperda* insect larvae (ca. 25%) compared to the contribution from soil or plant sources (< 5%). However, a large proportion of the insect microbiome could not be attributed to the sampled sources and was instead attributed to unknown sources (ca. 50%). Surprisingly, we found little evidence for exchange of fungal taxa, with the exception of a *Fusarium oxysporum* and a *Cladosporium* sp. OTU, between larvae and the infested leaves on which they fed. Together, our results suggest that mobile insects such as *S. frugiperda* obtain their fungal symbionts from a variety of sources, not limited to plants and soil, but including conspecific insects and other unsampled environmental sources, and that transmission among insects may play an important role in acquisition of fungal symbionts.

**Supplementary Information:**

The online version contains supplementary material available at 10.1007/s00248-022-02140-3.

## Introduction

Unlike sessile plants, animals often move across different environments and exchange microbial symbionts through interactions with other host species and their environment [1]. However, relatively few studies have examined the sources of microbial symbionts of mobile hosts such as insects [2, 3] or determined the degree to which insects exchange fungal symbionts with their environment, such as the plants on which they feed [4–6]. The extent to which microbial communities are shared and exchanged across insect hosts, plant hosts, and the soil environment has important implications for transmission of pathogens from pest insects to crops, as well as the efficacy of microbial biocontrol applications for these pests.

Herbivorous insects may acquire their fungal symbionts from a variety of environmental sources (e.g., soil, air, and water) or from interactions with other organisms (e.g., plant hosts). Results from one of the few studies of fungal microbiomes of foliar feeding insects showed that these insects acquire some of their fungal symbionts from the plants on which they feed [7]. However, in other systems, bacteria that typically inhabit the soil are acquired each generation and form symbiotic associations with herbivorous insects even though those insects feed only above ground [8]. Similarly, a recent study demonstrated that a leaf-feeding caterpillar (*Mamestra brassicae*) obtained a larger portion of its microbiome from soil rather than from the host plant on which it feeds [2].

Foliar feeding insects may also impact the microbial communities in their plant hosts [9]. The damage caused by insect herbivory to host plants creates routes of entry for fungi [10], and insects can also directly transmit fungi to plants [6]. The transmission of microbial symbionts from insects to plants can have negative consequences for plant host productivity through transmission of plant pathogens [6, 11, 12]. Thus, understanding the extent to which insects transmit fungal symbionts to plant hosts is important for maintaining agricultural plant health.

The fall armyworm (*Spodoptera frugiperda*) is a significant agricultural pest that feeds upon a wide range of crop species but shows a preference for and causes the greatest damage in various grasses, including corn (*Zea mays*), sorghum (*Sorghum bicolor*), and rice (*Oryza sativa*) [13]. Although *S. frugiperda* lays its eggs on leaf surfaces, where larvae hatch and cause substantial damage to plants by consuming foliage as they mature, pupation occurs in soil prior to emergence as adults each spring [14]. In its native range in North America, *S. frugiperda* migrates annually from overwintering grounds in south Texas and Florida, moving up to 90 km per week to expand northward during summer months through the midwestern and eastern U.S.A., respectively [15]. Recently, *S. frugiperda* has invaded and become a widespread and serious pest on several other continents, including India, Africa, and Asia, with devastating impacts on economics and food security [16–18]. Estimated costs of up to $13 billion can be attributed to *S. frugiperda* in Africa alone [19]. Knowledge of the sources of its fungal microbiome will help advance the use of microbes for management of this invasive pest.

To better understand sources from which herbivorous insects such as *S. frugiperda* acquire their fungi and whether these insects may exchange fungal symbionts with their plant hosts, we characterized fungal symbiont communities associated with *S. frugiperda* and those of four different ecological compartments in the insects’ immediate environment: *S. frugiperda* larvae feeding on sorghum, both infested and uninfested leaves of sorghum (*Sorghum bicolor*), and the soil environment surrounding *S. frugiperda*-infested plants. Using these datasets, we addressed the following questions. (Q1) How are fungal communities structured across co-occurring insect, plant, and soil ecological compartments? (Q2) What are the relative contributions of the source communities in these different ecological compartments to the fungal communities of insects? (Q3) Does the insect, *S. frugiperda*, contribute fungal taxa to the communities of the plant host on which it feeds?

## Methods

### Sample Collection and Processing

Samples were collected from four *S. frugiperda*-infested sorghum fields in Kansas, U.S.A., in July 2018. The *S. frugiperda* larvae were identified using the characteristic inverted Y-shaped marking on the head and four raised spots on the eighth abdominal segment [20]. Sampled insects were at the 4 to 6 instar stages. While it is known that the gut microbiome of insects may change during developmental stages or in response to diet [21, 22], these larval stages represent more mature stages that share a similar morphology and have fed on leaves during early development, but not yet pupated [14]. All sampling points were at least 3 m apart, and at each sampling point, we obtained one set of samples from each of four ecological compartments: a single *S. frugiperda* larva (insect), the leaf on which it was feeding (infested leaf), a leaf from the same plant without indications of *S. frugiperda* damage (uninfested leaf), and the soil beneath the plant (soil). Soil was sampled from the surface to a depth of 15 cm [23, 24], as close to the base of the plant as possible using a trowel, which was wiped with 70% ethanol between samples. In each field, 6–10 sets were sampled, for a total of 32 sample sets. All samples were stored individually in sterile whirl bags on ice until being processed, up to 24 h after collection.

Insect and plant samples were split in half and surface sterilized by rinsing in water, washed in 75% ethanol for 1 min, washed in 50% commercial bleach for 1 min, washed in 75% ethanol for 1 min, and then rinsed in sterile distilled water for 1 min [20, 25], with half of each sample frozen in liquid nitrogen immediately after returning the samples from the field for NextGen amplicon sequencing and the other half used for culturing. The microbiome of the entire insect was characterized in order to capture both gut microbes and those circulating in hemolymph. Due to time constraints, fungal cultures were attempted for only 10 sets of samples across the four fields. To do so, after surface sterilization, plant leaf samples were sectioned into 20 4 × 4 mm sections and plated onto ½ strength corn meal agar (CMA) [26] in 1.5 mL Eppendorf tubes (mini slants). The corresponding insects from the same 10 sets were sectioned into quarters and plated on ½ strength CMA plates. As fungi emerged from insects, individual colonies were transferred to ½ CMA slants.

### DNA Extraction, Amplification, and Sequencing

DNA was extracted from insect samples using a modified hexadecyltrimethylammonium bromide (CTAB) protocol [27, 28]. Frozen insect samples were lyophilized for 48 h and then ground to a fine powder using liquid nitrogen and sterile mortar and pestles. For each sample, homogenized insect tissue (10 mg) was added to 500 *µ*L of a 2% CTAB solution [27] amended with 66 *µ*L 70% sodium dodecyl sulfate (SDS) and 1.74 *µ*L of proteinase K (60 mg/mL), incubated at 65 °C for 3 h [28], and then extracted first with 666 *µ*L and a second time with 333 *µ*L 24:1 chloroform isoamyl alcohol. The purified aqueous fraction was treated with 5 *µ*L RNAse A (10 mg/mL) for two hours at 37 °C. DNA was precipitated with 460 *µ*L of isopropanol incubated at − 20 °C for one hour, centrifuged at 14,000 rpm for 5 min, and the DNA pellet washed with cold 70% ethanol and resuspended in 50 *µ*L 1% TE buffer. Total genomic DNA was extracted from plant tissue with the DNeasy Plant Mini Kit (QIAGEN, Valencia, CA, USA) using a volume of 200 *µ*L of plant tissue ground to a fine powder using liquid nitrogen and sterile mortar and pestles. Individual soil samples were homogenized using a coffee grinder sterilized with 70% ethanol between samples [29] and total DNA extracted from 10 mg of ground soil using the PowerSoil DNA Isolation Kit (QIAGEN, Valencia, CA, USA).

To generate amplicon sequencing data, the concentrations of extracted DNA samples were determined via Qubit fluorometer (Invitrogen, Carlsbad, CA), diluted to ca. 20 ng/µL, and 20 *µ*L per sample submitted to University of Minnesota Genomics Center (UMGC Microbiome Services, St. Paul, MN). The Internal Transcribed Spacer 1 region (ITS1F, ITS2 primers) of the rDNA SSU was amplified using a dual-index, two-step amplification method [30] and sequenced with a paired-end (2 × 250 bp) MiSeq 600 cycle kit in one lane (Illumina, San Diego, CA, USA).

To identify cultured fungi, total DNA was extracted from a small fungal tissue sample using the RED Extract’n’Amp Tissue PCR kit protocol (Sigma-Aldrich, St. Louis, MO, USA). The ca. 700 bp region of the rDNA locus was amplified by PCR using the ITS1F (SSU) and LR3R (LSU) primers [31, 32]. Successful amplification of DNA was confirmed via gel electrophoresis. DNA products were purified using the ExoSap-IT Product Cleanup Reagent protocol (Thermo Fisher Scientific, Waltham, MA, USA), and single-strand ITS1 sequences were obtained with Sanger sequencing using the ITS1F primer (GeneWiz, South Plainfield, NJ, USA).

### Bioinformatic Data Processing

ITS1 sequences obtained from MiSeq amplicon sequencing were demultiplexed, and after removing primer sequences, contigs were assembled and trimmed to 225 bp. Trimmed sequences were clustered into operational taxonomic units (OTUs) at 97% sequence identity using the QIIME2 toolkit (https://qiime2.org) [33] at the Minnesota Supercomputing Institute (MSI, Minneapolis, MN, USA). The OTUs were assigned taxonomic identities using a consensus BLAST method in QIIME2 against the full UNITE + INSD 2017 dataset (https://unite.ut.ee/) [34]. OTU table and taxonomy files were exported, and all subsequent analyses were performed in R (https://www.r-project.org/) [35] unless otherwise specified. Mapping files with metadata for each sample were manually compiled in Excel.

DNA sequences obtained from cultures were processed in Geneious v. 5.5.6 [36]. Sequences with multiple overlapping chromatogram peaks were removed and the remaining sequences cleaned by trimming the ITS1F and LR3R primer sequences. Sequences were clustered into OTUs at 97% sequence identity using the QIIME2 toolkit and then assigned taxonomic identities using a consensus BLAST method against the full UNITE + INSD 2017 dataset [34].

### Community Richness, Evenness, Composition, and Nestedness

To compare fungal taxa detected by culturing versus amplicon sequencing, all genera detected by culturing were compared to all genera detected by amplicon sequencing using the “get_taxa_unique” function of the “phyloseq” package in R [37]. The distribution and abundance of OTUs detected by both amplicon sequencing and culturing were plotted using the “plot_bar” function in the “phyloseq” R package [37].

The culture-independent amplicon sequencing results were used to characterize the fungal community structure of each ecological compartment (insect, infested and uninfested leaves, and soil). Sequencing depth for samples was compared using the “sample_sums” function in the “phyloseq” R package (https://joey711.github.io/phyloseq/index.html) [37] and visualized using the “rarecurve” function in the “vegan” R package (https://cran.r-project.org/web/packages/vegan/index.html) [38]. Sequence samples were not rarefied to a common number of sequences among samples, but rarefaction curves were constructed to estimate sampling sufficiency. Taxon richness in each fungal community was estimated as the observed number of OTUs using the “estimate_richness” function in the “phyloseq” R package [37]. Pielou’s evenness, which quantifies the skewness of taxon abundances within a community [39], was calculated using the “evenness” function in the “vegan” R package [38]. To assess differences among compartments for observed OTUs per sample and Pielou’s evenness, we performed separate linear mixed effect models using the “nmle” R package (https://cran.r-project.org/web/packages/nlme/index.html) [40] and performed subsequent post hoc Tukey tests in “base R” [35]. The full nlme mixed models included ecological compartment (compartment) as a fixed effect and field as a random effect (diversity ~ compartment, random =  ~ 1|field). The significance of the random effect term was assessed by fitting a reduced model with only the fixed effect term (diversity ~ compartment) and then performing an ANOVA using the “aov” function in base R [35] to compare the full and reduced models.

Differences in fungal community composition between different ecological compartments were assessed as abundance-weighted Bray–Curtis (BC) distances [41] using the “distance” function in the “phyloseq” R package with the “Bray” method [37]. The “adonis” function in the “vegan” R package [38] was used to perform a nested permutational multivariate ANOVA (PERMANOVA, BC ~ compartment/field) to determine if there was significant variation in Bray–Curtis distances among fungal communities associated with ecological compartment (compartment) and spatial location (field). The “betadisper” function of the “vegan” R package [38] was used to test for variation in dispersion of fungal communities within compartments and among fields.

The extent to which the less diverse fungal communities (e.g., insects and plants) were subsets of, or nested within, the more diverse fungal communities in soil was estimated using the “nestednodf” function (Nestedness Ordered by Decreasing Fill (NODF)) of the “vegan” R package [38], which returns values between 1 (no nesting) and 100 (perfectly nested) [42]. The significance of the NODF statistic was evaluated using the “oecosimu” function with 99 permutations in R v4.0 [43]. The occurrence of OTUs ordered by marginal totals was visualized using the “ComplexHeatmap” R package (https://bioconductor.org/packages/release/bioc/html/ComplexHeatmap.html) [44]. Samples were ranked by total number of OTUs occurring in that sample (row totals), and average ranks of each ecological compartment were analyzed using a Kruskal–Wallis test [45] implemented with the “kruskal” function in the “agricolae” R package (https://cran.r-project.org/web/packages/agricolae/index.html) [46].

To determine if the occurrence of fungal taxa in insects was predicted by their abundance (as read counts) in soil, for each of the 10 most commonly observed OTUs shared between insects and soil, we conducted a logistic regression of soil abundance and insect occurrence for paired soil and insect samples within replicate sample sets (glm(OTU occurrence in insects ~ OTU abundance in soil), binomial distribution (base R)) [35]. We defined the core insect microbiome as those OTUs present in 50% or more of the sampled insects and showing a relative abundance of at least 0.05% in each sample in which they were detected [47].

### FEAST Analyses

To estimate relative contributions of potential sources of symbionts to fungal communities in insect and plant compartments, we used the FEAST package (https://github.com/cozygene/FEAST) [48]. FEAST allows the user to designate microbial communities a priori as sinks or sources and then uses a maximization-estimation algorithm to attribute a percentage of taxa in the sink community to designated source communities based on similar patterns of occurrence and abundance of taxa between source and sink communities. Attribution of unknown sources occur in FEAST when a mismatch of taxon occurrence and abundance between the sink and source communities prevents assignment to a potential source, and thus, the sum of designated source contributions is less than 100%. The FEAST software currently does not allow identifying the specific taxa contributing to the unknown category or the designated sources.

Two types of FEAST analyses were performed: (1) a “leave-one-out” analysis to estimate average contribution from each potential source to the designated sink and (2) a “within-set” analysis, to estimate source contributions from the local environment associated with each sample set. For the “leave-one-out” analysis, all the sampled communities from the same ecological compartment were pooled as source communities. To consider the insect communities as sources for each individual insect sink, the target individual insect sink was left out of the pooled insect compartment source. For the “within-set” analysis, each sampled insect was designated as a sink, and the individual fungal communities in the infested leaf, uninfested leaf, and soil compartments within the same sample set were designated as potential sources. We also estimated the extent to which insects might contribute fungal symbionts to infested leaves by performing another “within-set” FEAST analyses treating each infested leaf as a sink and the uninfested leaf, insect, and soil compartments within the same sample set as potential sources.

## Results

From a total of 128 samples (4 ecological compartments per set, 32 sample sets across four fields), amplicon sequencing results were excluded for five sets due to poor sequence recovery for the insect samples (< 100 reads). For the remaining 27 sets, we recovered 6,769,278 reads, of which 4,072,065 passed quality control filters. On average, soil samples yielded the most reads (41,997 (SD ± 12,168)), followed by uninfested leaf samples (34,363 (SD ± 8135)), infested leaf samples (32,779 (SD ± 10,824)), and the insect samples (9117 (SD ± 12,340)). Sequences were binned into 3007 OTUs with 97% similarity of the ITS1 region using QIIME2. Rarefaction curves for fungal communities in each ecological compartment leveled off, indicating sampling depth was adequate (Fig. S1). Almost all cultured fungal genera were also detected by amplicon sequencing (Fig. S2). Statistical evaluation of cultured fungal communities was not conducted because the average number of cultures obtained per sample was low (2.2 per insect, 4.6 per infested leaf, and 6.1 per uninfested leaf).

### Fungal Community Structure and Composition in Different Ecological Compartments

**Fig. 1:**
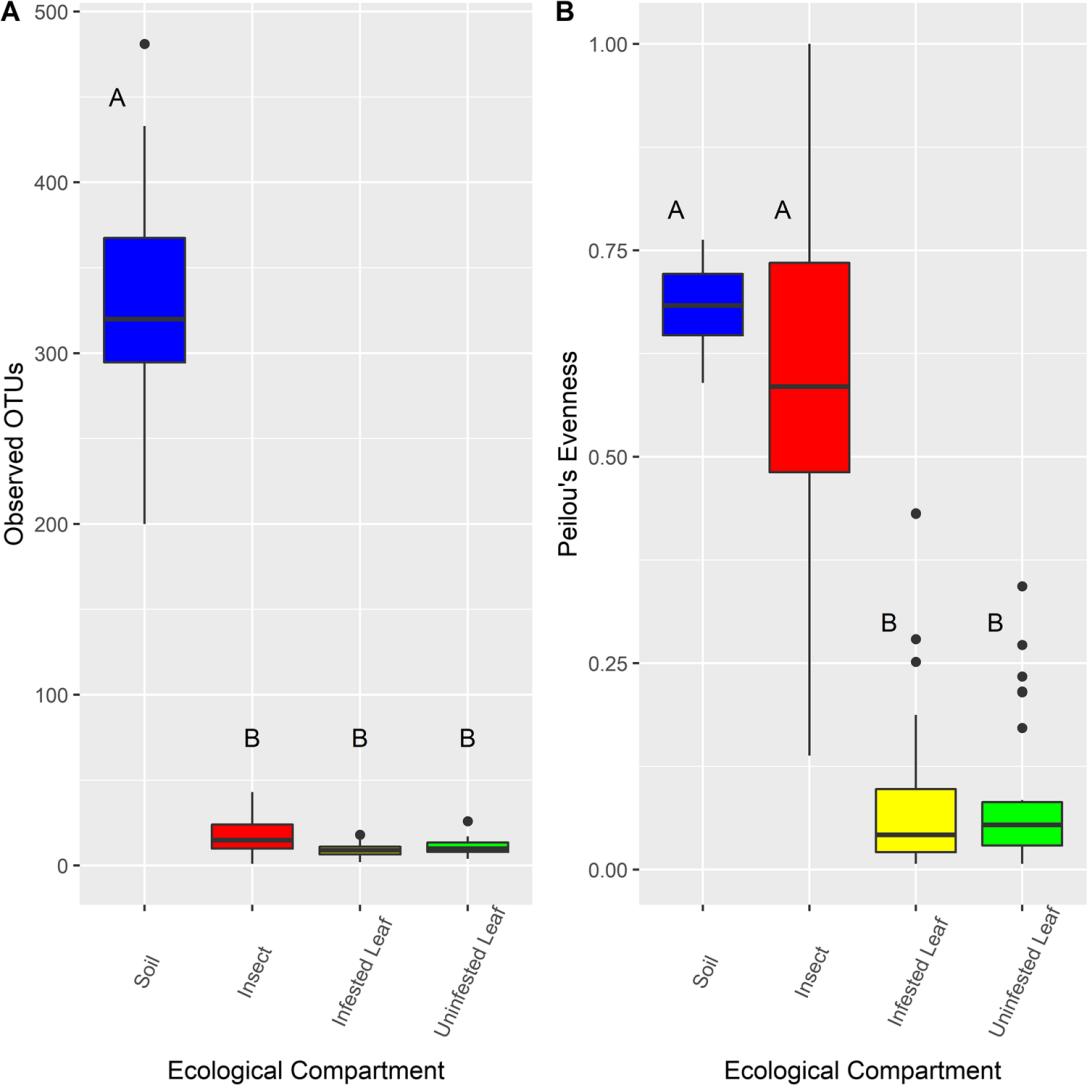
Richness and evenness of fungal communities in different ecological compartments. (**a**) Box and whisker plot of the number of observed OTUs per sample in fungal communities of soil, insect, infested leaf, and uninfested leaf compartments. The number of observed OTUs per sample differed significantly among ecological compartments (LME, *p* < 0.001; Table 1). Post hoc Tukey pairwise analyses found soil fungal communities had greater numbers of observed OTUs per sample than the fungal communities of insects, infested leaves, and uninfested leaves (letters above boxplots). (**b**) Box and whisker plot of Pielou’s evenness of fungal communities in soil, insect, infested leaf, and uninfested leaf samples. Fungal community evenness differed significantly among ecological compartments (LME, *p* < 0.001; Table 1). Post hoc Tukey pairwise analyses found that evenness of fungal communities in infested and uninfested leaf samples was similar to each other but significantly lower (*p* < 0.001) than evenness in insect and soil fungal communities (letters above boxplots)

We compared fungal community richness (observed OTUs per sample), evenness (Pielou’s Evenness), and composition (Bray–Curtis distance) in the different ecological compartments. Variation in observed OTUs per sample was significantly affected by ecological compartment (*p* < 0.001, LME (observed ~ compartment, random =  ~ 1|field)) and the spatial factor of field (*p* = 0.02) (Table 1). Subsequent post hoc Tukey pairwise tests revealed that soil fungal communities had significantly more (*p* < 0.001) observed OTUs per sample than did communities in other ecological compartments (Fig. 1a). Results for Pielou’s evenness (LME (evenness ~ compartment, random =  ~ 1|field)) showed that ecological compartment, but not field, contributed significantly to variation in evenness of fungal communities (*p* < 0.001; Table 1). Subsequent post hoc Tukey tests showed that fungal communities in infested and uninfested leaves showed significantly lower evenness than fungal communities in soil (*p* = 4.96 × 10^−14^) and insects (*p* = 4.96 × 10^−14^) (Fig. 1b), likely due to the high abundance of one taxon, *Cladosporium* sp. 1 (OTU 2348) in both infested and uninfested leaves (Fig. S2a). The variation among insect fungal communities for Pielou’s evenness tended to be larger than for other ecological compartments (Fig. 1b). Consistent with high variation in evenness observed in insect communities, we found only five OTUs that might be considered core taxa, those present in 50% of more of insects sampled (prevalence) and with greater than 0.05% abundance per insect in which it occurred [45] (Table S1). Of these, only three taxa, *Alternaria alternata* (OTU 1549), *Cladosporium chasmanthicola* (OTU 1851), and *Fusarium cuneirostrum* (OTU 2481), showed an average relative abundance per insect greater than 10%.

**Table 1:** Results of linear mixed model (lme; dependent variable ~ compartment, random =  ~ 1|field) analysis examining the effect of ecological compartment (insect, infested leaf, uninfested leaf, and soil) on observed OTUs per sample and Pielou’s evenness of communities within samples

Dependent variable	Observed OTUs					Evenness				
Effect	Df	Sum of squares	MeanSqs	*F* value	*p* value	Df	Sum of squares	MeanSqs	*F* value	*p* value
Intercept	1	101,587	986	191.4	< 0.001^*^	1	1.960	0.019	824.4	< 0.001^*^
Compartment	3	2,059,575	686,525	696.4	< 0.001^*^	3	8.5	2.8	152.6	< 0.001^*^

^*^ANOVAs comparing full (above) and reduced (dependent variable ~ compartment) models show significant variation of observed OTUs due to field (*p* = 0.02) but does not show significant variation in evenness due to field (*p* = 0.99)

**Fig. 2:**
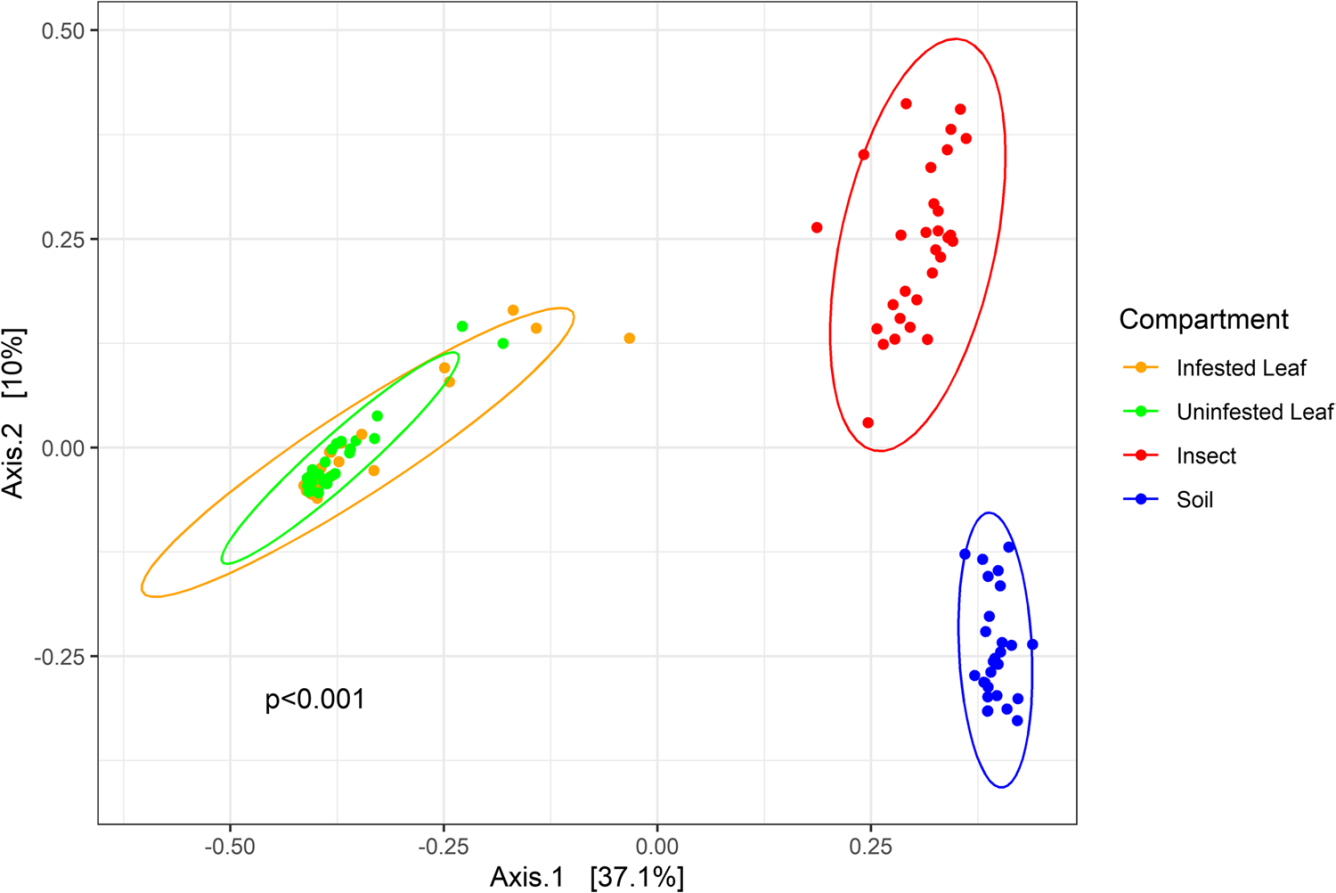
PCoA of Bray–Curtis distances among fungal communities of different ecological compartments. Community composition in infested and uninfested plant leaves largely overlapped, whereas PERMANOVA of Bray–Curtis distances found significant differences in composition of fungal communities among ecological compartment (*p* < 0.001), likely due to the differences among plant, insect, and soil samples (Table 2)

Analyses of community composition showed significant differences in Bray–Curtis distances among fungal communities in insect, soil, and plant compartments (*p* < 0.001, PERMANOVA (BC ~ compartment/field)), but little difference in Bray–Curtis distances among communities of infested and uninfested leaves (Table 2). Variation across fields also contributed significantly to variation in Bray–Curtis distances (*p* < 0.001; Table 2). The results of a Principal Coordinates Analysis (PCoA) of Bray–Curtis distances also showed that fungal communities from insects, plants, and soil were distinct, while those within plants (infested versus uninfested leaves) were overlapping and highly similar (Fig. 2).

**Table 2:** Results of PERMANOVA on Bray–Curtis distances as a response to compartment, nested within field (BC ~ compartment/field). Ecological compartment (compartment) and spatial effects (field) were significantly associated with variation in a Bray–Curtis distances

Term	Df	Sum of squares	MeanSqs	*F* value	*p* value
Compartment	3	17.5	5.8	32.9	< 0.001^*^
Compartment/field	12	4.5	0.4	2.1	< 0.001^*^

**Fig. 3:**
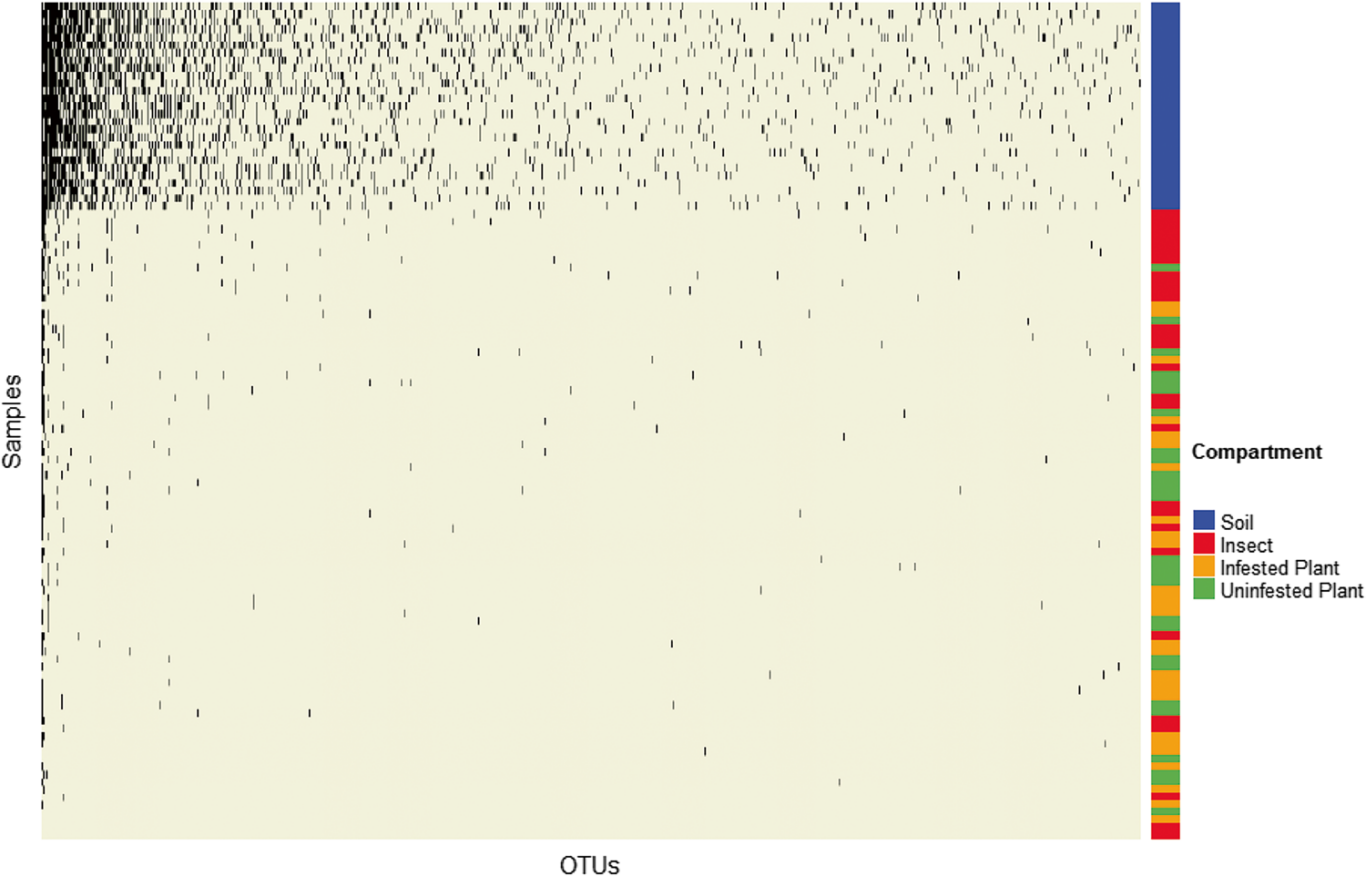
Nestedness of fungal communities from different ecological compartments. Columns represent the occurrence of individual OTUs (taxa) in each sample and are ordered by total occurrence across samples (column totals) from greatest to least (left to right). Rows represent the occurrence of observed OTUs in individual samples from different ecological compartments and are ordered by the total number of observed OTUs (row totals) from greatest to least (top to bottom). The ecological compartment from which samples were obtained is shown in the column to the right (blue = soil, red = insect, orange = infested leaf, green = uninfested leaf). The overall Nestedness Ordered by Decreasing Fill (NODF) statistic was small but significant (NODF = 15.45, *p* = 0.01). NODF_columns_ (OTU occurrence in samples) contributed significantly to nestedness (*p* = 0.01), but NODF_rows_ (numbers of OTUs in samples) did not (*p* = 0.39)

As we observed that OTU (species) richness was greatest in soil communities, followed by those in insects, and finally those in plants, we used a nestedness analysis [42, 43] to evaluate the extent to which communities occupying living hosts (insects and plants) might be subsets of those residing in the soil. Results showed that the mean value of the nestedness statistic, NODF, was small but significantly different than 1 (NODF = 15.45 on scale of 1 to 100, *p* < 0.01), suggesting a low level of nestedness (Fig. 3). However, consistent with the Bray–Curtis results above, the columns (NODF_columns_, number of different taxa) significantly contributed to NODF (*p* < 0.01), whereas rows (NODF_rows_, total numbers of OTUs/individual sample) did not, suggesting that compositional turn-over, more than nestedness, contributes most to differences among these communities (Fig. 3) [49]. Consistent with the results for OTU richness, there were significant differences in rank order of richness (row totals of OTUs/individual sample) in samples representing the different ecological compartments (Kruskal Wallis test; Table S2). Soil fungal communities ranked as the most diverse, followed by fungal communities in insects, and then fungal communities in infested and uninfested leaves. Together, the results of the richness, Bray–Curtis, and nestedness analyses show that the less diverse fungal communities in insects were distinct from, but not strongly nested within, the taxa present in soil.

Nonetheless, we found that ca. 50% of fungal taxa were shared between soil and insect ecological compartments. We performed logistic regressions with the expectation that if insects obtain these taxa directly from soil, then taxa that are more abundant in soil samples should also occur more frequently in the associated insect samples. We did not find significant correlations for any taxon, suggesting that insects do not directly acquire many fungal symbionts from the soil (Table S3).

### Estimated Source Contributions to Fungal Communities in Insects

**Fig. 4:**
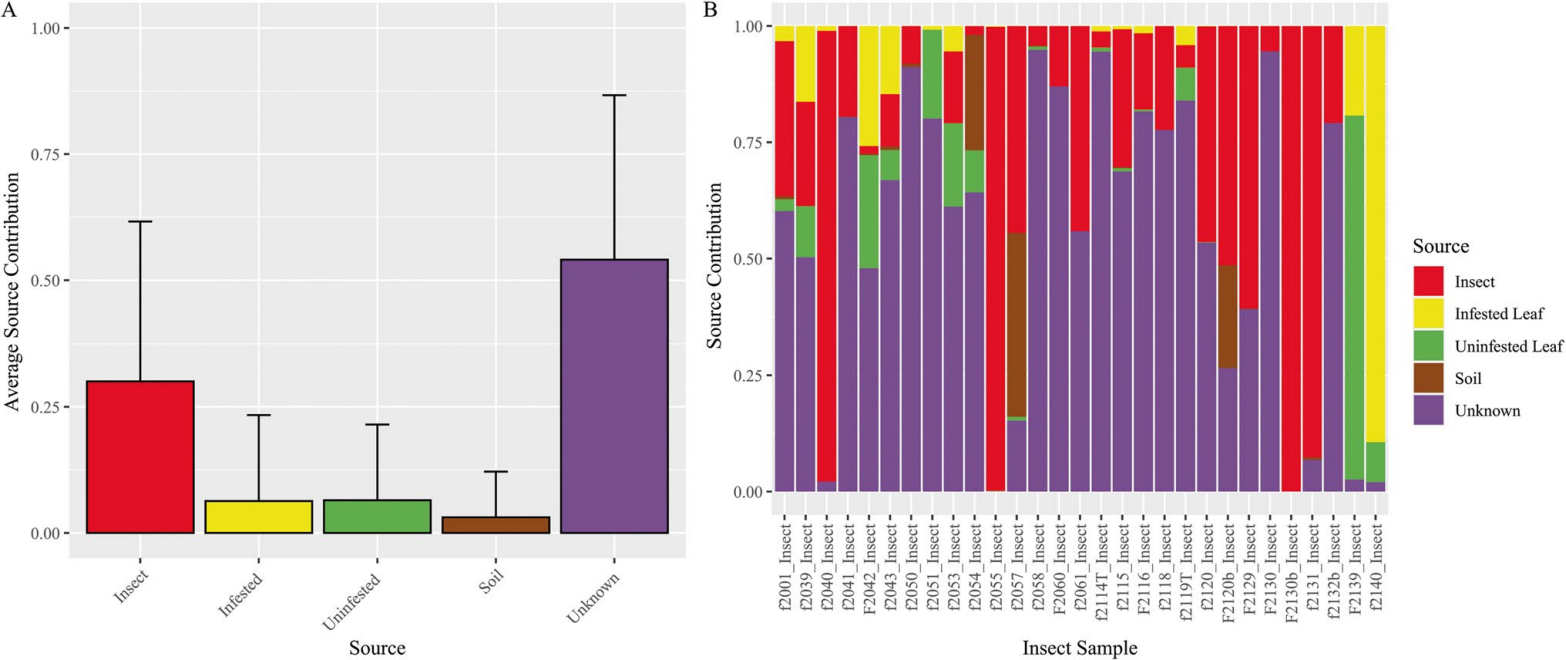
Results of “leave-one-out” FEAST analyses. To estimate the relative contribution from potential source communities (ecological compartments) to fungal communities in insects, fungal communities in individual insects were designated as sinks and the fungal communities in soil, infested leaf, uninfested leaf, and remaining insects were pooled by ecological compartment and designated as sources. (**a**) Average source contributions to fungal communities in insects. Of the designated sources, other insects were attributed as a source for 30% (SD ± 31.6) of the fungal communities in insects, while soil (3.1% SD ± 9.1) and plant (infested 6.3% SD ± 17; uninfested 6.5% SD ± 15) sources contributed a much lower percentage to fungal communities in insects. A large proportion (54% SD ± 32.6) of the fungal communities in insects were attributed to unknown sources. (**b**) Estimated source contributions to fungal communities in individual insect samples. Each bar on the *x*-axis represents the fungal community in an individual insect, with the estimated relative contributions of designated pooled or unknown sources shown on the *y*-axis. Source contributions estimated by “leave-one-out” FEAST analyses were highly variable among individual insect fungal communities

We used the FEAST statistical package to estimate the relative contributions of fungal communities in each ecological compartment (designated as sources) to the fungal communities in insects (designated as sinks). We first conducted a “leave-one-out” analysis in which all samples for each potential source compartment were pooled, but the insect sample pool left out the individual insect sink under analysis. The results of the “leave-one-out” analyses showed that on average, FEAST attributed the pooled insect source communities as contributing a larger fraction of symbionts (30% SD ± 31.6) to the individual insect sinks, compared to the other designated source communities (soil, 3.1% SD ± 9.1; infested leaves, 6.3% SD ± 17; and uninfested leaves, 6.5% SD ± 15) (Fig. 4a). Interestingly, over half of the individual insect sink communities could not be attributed to one of the designated sources and were instead attributed to unknown sources (54% SD ± 32.6; Fig. 4a). There was substantial variation among individual insect sinks in the fraction of the fungal community attributed to each source (Fig. 4b). For example, fungal communities in four individual insects (f2040, f2055, f2130b, and f2131; Fig. 4b) were attributed almost completely to other insect sources, the fungal communities in two individual insects (f2139, f 2140; Fig. 4b) were mostly attributed to infested or uninfested leaf sources, and those in eleven of the individual insect sinks were primarily attributed to unknown sources (f2041, f2050, f2051, f2058, f2060, f2114T, f2116, f2118, f2119T, f2130, and f2132b; Fig. 4b).

**Fig. 5:**
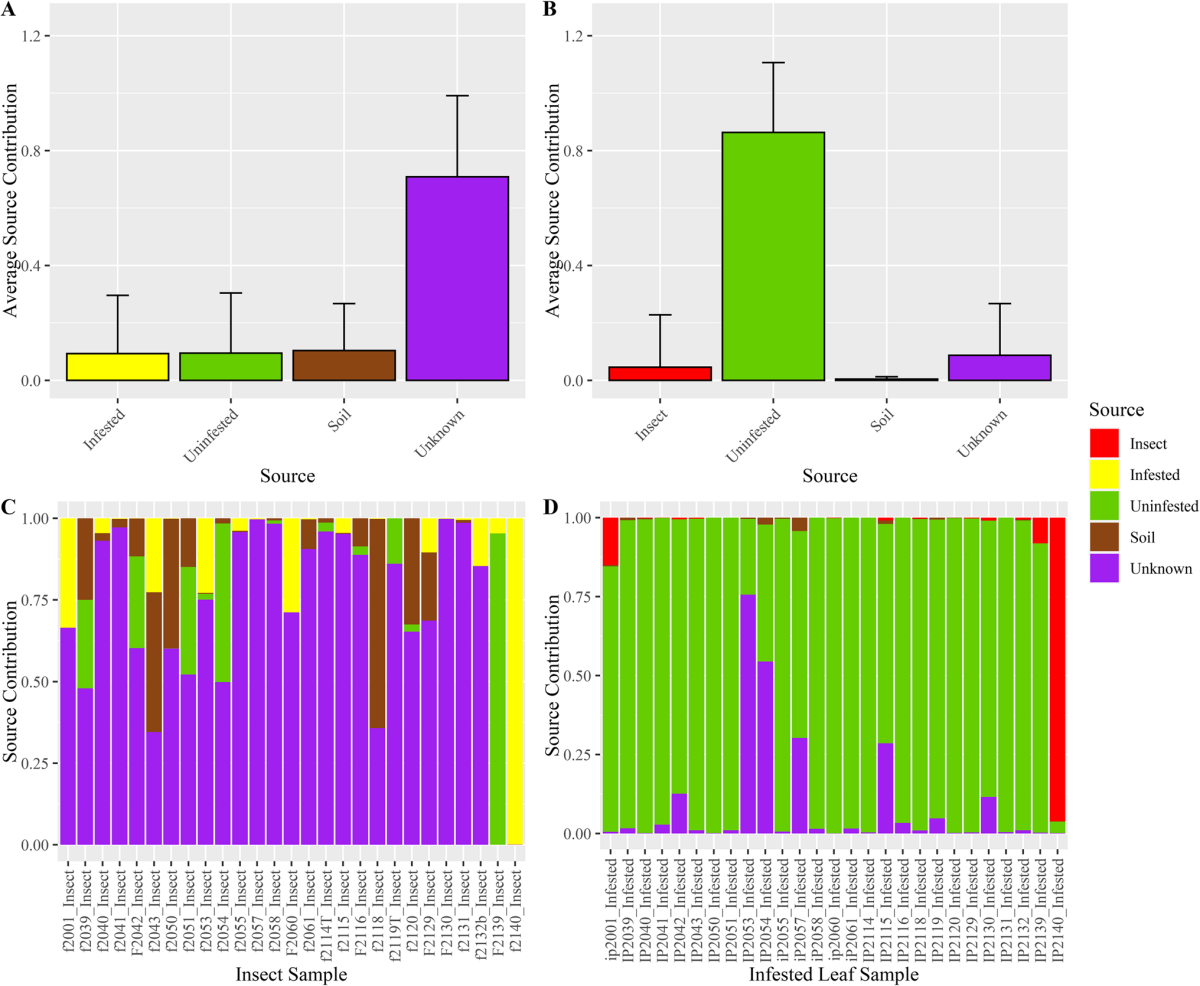
Results of “within-set” FEAST analyses. To estimate contributions from environmental sources in the immediate proximity of insects or to the infested leaf on which the insect fed, each individual insect or infested leaf was designated as a sink and the remaining ecological compartments within the same sample set (soil, infested leaf, and uninfested leaf for insects; insects, uninfested leaf, and soil for infested leaves) were designated as sources. (**a**) Average source contributions to fungal communities in insect samples. The “within-set” FEAST analyses attributed similar percent contributions from sources of soil (10.3%, SD ± 16.4), infested leaf (9.3%, SD ± 20.2), and uninfested leaf (9.5%, SD ± 20.9) to fungal communities in insects. A larger proportion were attributed to unknown sources (70.8%, SD ± 28.3) than in the results of “leave-one-out” analyses. (**b**) Average source contribution to fungal communities of infested leaf tissues. On average, a majority of fungal communities of infested leaves (86.3%, SD ± 24.4) were attributed to uninfested leaves, likely due to high similarity between these communities. In contrast, insect sources were rarely attributed (5%, SD ± 18.2) to the fungal communities of infested leaves. (**c**) Estimated source contributions to fungal communities of individual insects. Each bar on the *x*-axis represents a fungal community in an individual insect, with the estimated relative contributions of designated sources or unknown sources along the *y*-axis. Source contributions estimated by “within-set” FEAST analyses were variable among individual insect communities but dominated by unknown sources with negligible contribution attributed to infested leaves. (**d**) Estimated source contributions to individual fungal communities in infested leaf samples. Each bar on the *x*-axis represents a fungal community in an individual infested leaf sample with the estimated relative contributions of designated “within-set” and unknown sources along the *y*-axis. Insect sources were rarely attributed to the communities of infested leaf samples, consistent with the results above, and with the “leave-one-out” analysis

To ask whether the large amount of variation in source contributions among individual insects that we note above might be due to variation across local environmental sources, we also performed FEAST analyses within each sample set at each sampling location, assigning the individual insect community as the sink and communities from other compartments within the same sample set as sources. Results of this “within-set” FEAST analyses showed that, on average, only small fractions of the fungal communities in insect sinks could be attributed to the fungal communities of the infested leaf (9.3% SD ± 20.2), uninfested leaf (9.5% SD ± 20.9), or soil compartment (10.3% SD ± 16.4) in the same set and sampling location, and most were instead attributed to unknown sources (70.8% SD ± 28.3; Fig. 5a). The unknown contribution estimated in the “within-set” FEAST analyses was likely inflated relative to the “leave-one-out” analyses because insects could not be included as a source. Surprisingly, fungal communities in infested leaves were not attributed as a source more frequently than were uninfested leaves even though the sampled insect was in contact with and consuming the infested leaf (Fig. 5a). Again, individual insect sinks exhibited substantial variation in source contributions, suggesting acquisition of symbionts from different sources encountered by these insects in their environment (Fig. 5c).

### Estimated Contribution of Insect Sources to Fungal Communities in Infested Leaves

To assess the extent to which insects may contribute to the fungal communities of the infested leaves they reside on, we also performed “within-set” FEAST analyses using fungal communities of infested leaves as sinks and communities from other compartments within the same set as sources. Insect sources were not commonly attributed as a source for fungal communities in infested leaves (4.6% SD ± 18.2, Fig. 5b). Instead, most fungal communities of infested leaves were attributed to the uninfested leaf source (86.3% SD ± 24.4), which is likely due to the similarity of fungal communities in infested and uninfested leaves (Figs. 5b, d).

To identify specific taxa that might have been exchanged between insects and plants, we identified individual OTUs observed in both the insect and the paired infested leaf, but not in the uninfested leaf within the same sample set. We found only 15 cases fitting these criteria (Table S4). In 6 of these 15 cases, a *Fusarium oxysporum* OTU (OTU_671) was shared, and in 3 cases, *Cladosporium* sp. 2 (OTU_701) was shared. Interestingly, these two taxa were often found in insects (Table S1), suggesting that they are common in the environment of both the insects and the plants. In each of the remaining 6 cases, a different OTU was represented, suggesting that these were uncommon taxa not well sampled by our methods. Overall, these and the FEAST results above suggest that aside from these *Fusarium* and *Cladosporium* OTUs, insects exchange very few fungal symbionts with leaves on which they are feeding.

## Discussion

We describe the fungal communities in four different ecological compartments (insects, infested leaf, uninfested leaf, and soil) and estimate the exchange of fungal symbionts among insects and these other compartments. We report three key findings. First, fungal community composition differed among soil, leaf, and insect samples, while the composition of infested and uninfested leaves of the same plant was very similar. Fungal communities in insects and plants harbored far fewer taxa than did soil communities but were not strongly nested within the richer soil communities. Second, the results of a “leave-one-out” FEAST analyses showed that other *S. frugiperda* insects were attributed as sources of the fungal communities in individual insects more commonly than the soil and plants sources. Along with our observation of considerable variation among individual insect sink communities in the sources attributed by FEAST, including substantial contributions attributed to unknown sources, our results suggest that *S. frugiperda* acquires fungi from a variety of environment sources, not only from plants and soil, but also from other *S. frugiperda* insects and environmental sources not sampled. Third, while few fungal taxa are apparently exchanged between the insect and the plant on which the insect feeds, *Fusarium oxysporum* and *Cladosporium* sp.2 OTUs apparently are and could be important to plant health. Our results provide insights into the sources that contribute most strongly to shaping the composition of fungal microbiomes in herbivorous insects and in the host plants on which they feed.

Ecological compartments of soil, plants, and insects harbored distinct communities that differed in diversity and evenness of fungal taxa. Compared to the fungal communities in plants and insects, those in soil showed the greatest OTU richness and were affected by spatial location (field), a pattern observed in other studies of microbial communities across different ecological compartments [29, 50]. Fungal communities in infested and uninfested *S. bicolor* leaves exhibited lower OTU richness and evenness than did those in other compartments, likely because these communities were dominated by a single *Cladosporium* taxon (OTU 2348). Previous characterization of endophyte communities of *S. bicolor* leaves also found similarly low diversity due to an abundance of *Cladosporium* taxa [51]. Fungal communities of insects showed intermediate richness and tended to have greater variation in evenness among individuals, compared to fungal communities in plants and soil. High variation among microbial communities of different individual insects is common among other lepidopteran microbial symbiont communities [3, 25, 52, 53], leading some to hypothesize these communities are more transient, strongly affected by environmental sources with which they interact, and composed of few “core” taxa [25]. However, recent research has identified a core bacterial microbiome in the gut of *S. frugiperda* across different plant hosts and locations in South America [47] and we identified five taxa shared across a majority of individual insects sampled (Table S1). While these taxa may be candidates for more stable symbiont associations with *S. frugiperda*, distinguishing between transient and stable symbionts is not possible from next-generation sequencing data sampled at a single time point and will require further experimental studies.

In contrast to previous studies, our results did not suggest that these insects acquire a majority of their symbionts directly from the soil or plant environment. Not only was there little correlation between the abundance of the most common taxa in soil and their occurrence in associated insects (Table S3), but there were relatively few shared taxa between plants and insects (Table S4). Similarly, the FEAST analyses did not attribute a large proportion of the microbiome to soil or plant sources, but instead to other insect and unknown sources (Figs. 4, 5). The large attribution to unknown sources could be due to sources we did not sample, for example, the epiphytic fungal communities on the surface of leaves [54] or fungi carried in the air, or could result from the inability of FEAST to match the composition (identity and abundance of taxa) of communities in an individual insect to that of the pooled, designated sources [48]. Such mismatches could occur if insects acquire fungal taxa from designated sources but subsequently filter those taxa, resulting in different abundances of these taxa in insects. Plant hosts are known to filter fungi from the soil environment, such that only a subset of taxa encountered in soil are able to survive, grow, and compete successfully in the interior of the root, resulting in a highly similar root microbial communities [29, 55]. In contrast, we observed the microbial communities in these insects to be highly variable with few taxa shared among individual insects. Additionally, insect symbiont communities were not strongly nested within those of soil or plants, indicating instead high turn-over between ecological compartments [49]. Overall, our data suggest that mobile insect hosts such as *S. frugiperda* encounter and acquire symbionts from a greater number of sources they may interact with in their environment, than do their non-mobile plant hosts.

Results from the “leave-one-out” FEAST analyses indicate that *S. frugiperda* may acquire a substantial fraction of its fungal symbionts from other conspecific insects rather than from environmental sources such as soil [2] or plant hosts [7]. We infer that the strong signal of insect sources in the FEAST analysis is not primarily due to vertical transmission, which should lead to more homogenous and less diverse communities than we observe here [56]. Although our understanding of vertical transmission from parent to offspring among insects has been primarily derived from studies of bacterial symbionts [57–61], vertical transmission of fungal symbionts in insects does occur [56, 62–64]. Transovarial transmission was recently demonstrated as a mechanism for *Ophiocordyceps* fungal symbionts of scale insects [63], as well as for the microsporidian pathogen *Nosema bombyci*s in the related tobacco cutworm pest *Spodoptera litura* [65]. Although *S. frugiperda* may harbor a few taxa that are vertically transmitted, perhaps those we identify as core, and shared across a majority of insects sampled (Table S1), we infer that the strong source contribution of conspecific insects found by FEAST is more likely due to horizontal transmission, the acquisition of microbial symbionts from other insects or from shared environmental sources. Horizontal transmission is predicted to result in more variable and diverse microbial communities, with fewer taxa held in common among individuals [66], as we observe here. Close interactions between larvae inhabiting the same infested leaf, and in particular, cannibalism, could constitute mechanisms of horizontal transmission. Cannibalism is a documented behavior of *S. frugiperda* larvae both in the laboratory and the field and is known to transmit nuclear polyhedrosis virus among *S. frugiperda* larvae [67]. Further research using direct experimentation is needed to determine mechanisms by which this insect might acquire its fungal symbionts from other insects. Given the large attribution of unknown sources by FEAST to the fungal microbiome of *S. frugiperda*, as well as the high variability in both community evenness and source attributions by FEAST among individual insects, our results are best interpreted as showing that *S. frugiperda* acquires most of its fungal symbionts from a diversity of spatially varying sources, possibly including some that we did not sample.

In contrast to other studies demonstrating that phytophagous insects are competent vectors of fungal symbionts to plant hosts [4–6], we found surprisingly little evidence of exchange of fungal symbionts between the fall armyworm and infested sorghum plants. Additionally, we did not find fungal communities of uninfested plants to be nested within fungal communities of infested plants, as has been observed in other studies [9]. The only examples of potential exchange among insect and plant compartments included that of a *Fusarium oxysporum* taxon (OTU_617) and a *Cladosporium* sp. 2 taxon (OTU_701) found in both insects and their associated infested leaves, but not in the fungal communities of uninfested leaves of the same plant. Members of the *F. oxysporum* species complex are known to infect multiple hosts and have been detected as both endophytes and pathogens of plants [68], as well as pathogens of insects and animals [69, 70]. Additionally, sciarid (*Bradysia* spp., *Lycoriella* spp., and *Sciara* spp.) and shore flies (*Scatella* spp.) were previously shown to vector plant pathogenic strains of *F. oxysporum* among cucumber plants in a greenhouse study [71]. *Cladosporium* taxa are also documented as common endophytes of plants [72] and have also been found in the human gut [73] and as both beneficial symbionts and pathogens of insects [74, 75]. Consequently, while our results show that insect infestation does not substantially affect the fungal community structure of sorghum leaves and these insects likely do not gain many taxa from plants, the exchanges of *Fusarium* and *Cladosporium* taxa could affect plant and insect health.

We conclude that there are distinct fungal communities in *S. frugiperda*, its plant host *S. bicolor*, and the surrounding soil environment. Results suggest *S. frugiperda* likely acquires symbionts from a variety of sources in their environment, including a substantial contribution from conspecific insects, rather than primarily from the plants on which they feed or the soil. We found that the community structure of fungal symbionts of these migratory insects varied greatly from insect to insect, compared to variation across individuals of the sessile plant host. Surprisingly, there was relatively little contribution to the fungal microbiome of *S. frugiperda* from the plant hosts on which they fed and little evidence for transmission of fungal symbionts between the insect to its plant host. These results increase our understanding of the varied environmental sources of fungal taxa for the microbiome of this important insect pest and illustrate potential processes by which fungal microbiomes are structured across distinct, interacting host species.

## Supplementary Information

Below is the link to the electronic supplementary material.

Supplementary file1 (DOCX 104 KB)

## Data Availability

Raw sequencing data will be deposited into SRA prior to publication and representative OTU sequences from cultured isolates will be deposited into GenBank. Metadata are to be deposited into SRA with the raw sequence data.
