# Supplementary material for: Sources of Fungal Symbionts in the Microbiome of a Mobile Insect Host, Spodoptera frugiperda
Source: Microb Ecol. 2022 Dec 8;86(2):900–13. doi: 10.1007/s00248-022-02140-3 (PMC10335968; doi:10.1007/s00248-022-02140-3)
Supplement: Supplementary file 1 — Supplementary file1 (DOCX 104 KB) [file 248_2022_2140_MOESM1_ESM.docx]

**Table S1**. Insect core microbiome analysis: The taxonomic assignment, prevalence, and average relative abundance (across all samples) of those OTUs that occurred with greater than or equal to 50% prevalence and 0.05% relative abundance in all individual insects in which it was detected.

| OTU Taxonomic Assignment | OTU ID | Prevalence | Avg. Relative Abundance |
| --- | --- | --- | --- |
| *Alternaria_alternata* | 1549 | 75.00% | 23.43% |
| *Cladosporium_chasmanthicola* | 1851 | 75.00% | 13.46% |
| *Cladosporium sp. 2* | 701 | 67.86% | 3.64% |
| *Fusarium_cuneirostrum* | 2481 | 57.14% | 12.85% |
| *Fusarium_oxysporum* | 671 | 50.00% | 4.25% |

**Table S2.** Results of Kruskal-Wallis test on the rank order of sampled fungal communities ordered by decreasing number of observed OTUs per sample (row totals) from top to bottom. Average rank is listed with standard deviation. Group letter denotes significant differences in rank order of OTU richness among ecological compartments.

| Ecological Compartment | Average Rank | Group |
| --- | --- | --- |
| Soil | 96±7.9 | a |
| Insect | 53.75±26.2 | b |
| Uninfested Leaf | 38.63±20.0 | c |
| Infested Leaf | 31.67±19.6 | c |

**Table S3**. The results of logistic regressions correlating the occurrence of each of ten fungal OTUs in insects with the abundance of the same OTU in soil samples associated with each insect. These OTUs are the 10 most commonly observed OTUs across sets shared between soil and insects. Columns include the taxonomic assignment of the OTUs, the number of times the OTU occurred in insect samples across 27 sets, the range of sequence reads observed across the soil samples, and the reported p-value for the logistic regression. There were no significant relationships between the occurrence in insects and abundance in soil for any OTU.

| OTU taxonomic assignment | OTU ID | | # Detections in Insects | Soil Sequence Read Range | p-value |
| --- | --- | --- | --- | --- | --- |
| *Alternaria alternata* | 1549 | 20 | | 41-15,089 | 0.32 |
| *Cladosporium sp. 1* | 2348 | 26 | | 0-189 | 0.62 |
| *Cladosporium sp. 2* | 701 | 19 | | 0-115 | 0.47 |
| *Cladosporium chasmanthicola* | 1851 | 20 | | 9-1,934 | 0.99 |
| *Clonostachys sp.* | 751 | 7 | | 0-276 | 0.12 |
| *Filobasidium magnum* | 807 | 7 | | 0-1,402 | 0.67 |
| *Fungi (unclassified)* | 2874 | 9 | | 0-445 | 0.64 |
| *Fusarium cuneirostrum* | 2481 | 15 | | 0-115 | 0.30 |
| *Fusarium oxysporum* | 671 | 14 | | 0-2,061 | 0.97 |
| *Moesziomyces sp.* | 2240 | 12 | | 0-191 | 0.17 |

**Table S4.** OTUs observed in fungal communities of sampled insect and infested leaf within a set, but not observed in fungal communities in the uninfested leaf sample within the same set.

| OTU taxonomic assignment | OTU ID | # Sets | % of total sets |
| --- | --- | --- | --- |
| *Fusarium oxysporum* | 671 | 6 | 22.2 |
| *Cladosporium sp. 2* | 701 | 3 | 11.1 |
| *Fungi (unclassified)* | 1620 | 1 | 3.7 |
| *Tremellomycetes sp.* | 2693 | 1 | 3.7 |
| *Fungi (unclassified)* | 3722 | 1 | 3.7 |
| *Filobasidium magnum* | 807 | 1 | 3.7 |
| *Cladosporium chasmanthicola* | 1851 | 1 | 3.7 |
| *Moesziomyces sp.* | 2240 | 1 | 3.7 |


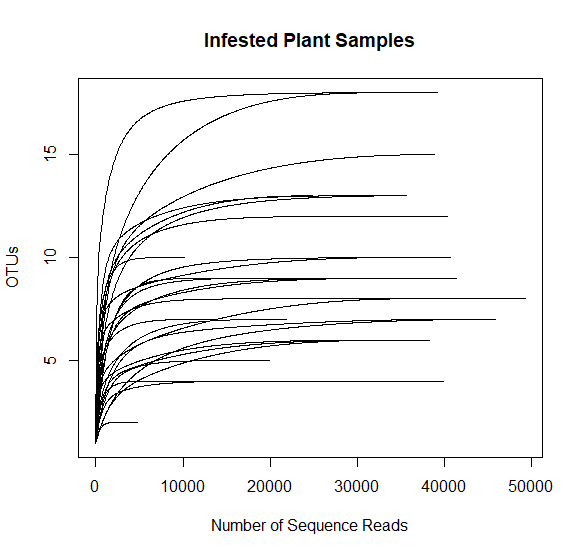

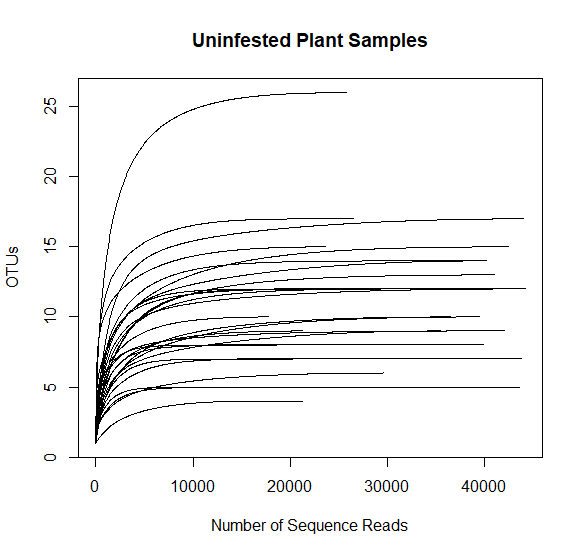

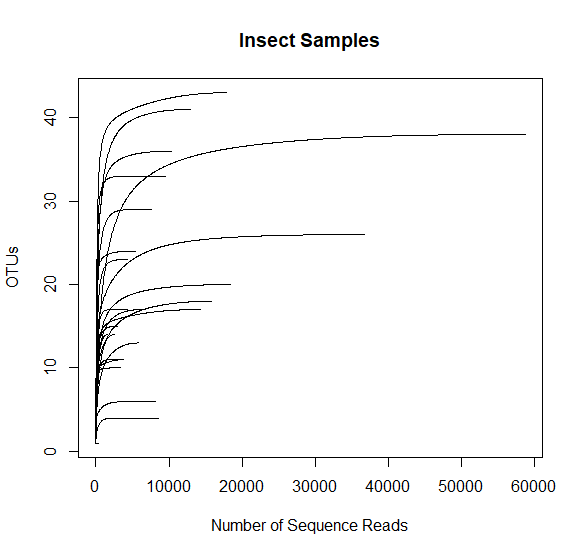

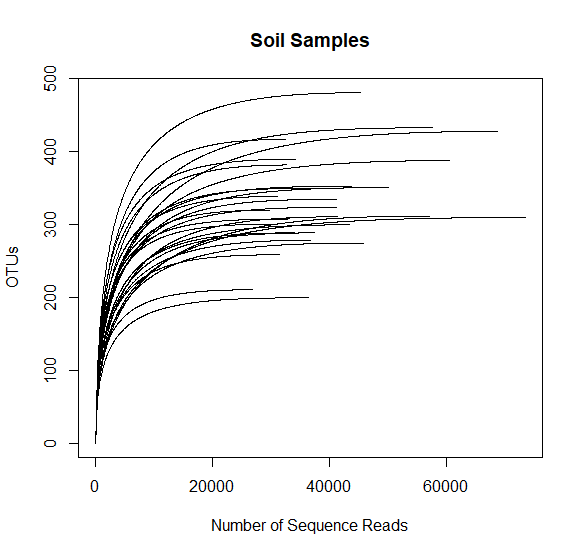


**Figure S1.** Rarefaction curves for fungal communities from different ecological compartments. All rarefaction curves leveled off, indicating sufficiently saturated sampling. Fungal communities in insect had the most variation and the lowest average number of sequence reads per sample.


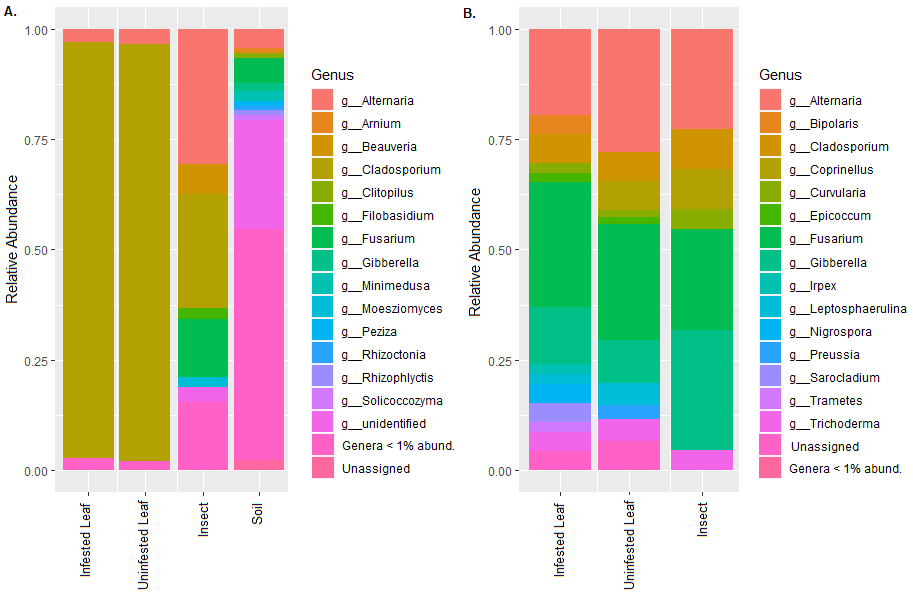


**Figure S2.** Abundance of fungal taxa in ecological compartments (infested leaf, uninfested leaf, insect, soil). **A)** Relative abundance of genera detected by amplicon sequencing in infested leaf, uninfested leaf, insects, and soil. Genera that made up less than 1% of the relative abundance were grouped are represented by “Genera <1%”. *Alternaria* is the most common genus shared between insect and soil ecological compartments. In the fungal communities of both the infested and uninfested leaf samples, a *Cladosporium* sp*.* OUT (OTU 2348) was the most abundant taxon. **B)** Relative abundance of genera cultured from infested leaf, uninfested leaf, and insects (soil was not cultured). Genera that made up less than 1% of the relative abundance were grouped and are represented by “Genera <1%”. The OTUs assigned to the genera *Coprinellus, Leptosphaerulina*, and *Trametes* were detected by culturing but not by amplicon sequencing.
